# uPAR Immuno-PET in Pancreatic Cancer, Aging, and Chemotherapy-Induced Senescence

**DOI:** 10.2967/jnumed.124.268278

**Published:** 2024-11

**Authors:** Edwin C. Pratt, Riccardo Mezzadra, Amanda Kulick, Spencer Kaminsky, Zachary V. Samuels, Angelique Loor, Elisa de Stanchina, Scott W. Lowe, Jason S. Lewis

**Affiliations:** 1 Department of Radiology, Memorial Sloan Kettering Cancer Center, New York, New York;; 2 Cancer Biology and Genetics Program, Memorial Sloan Kettering Cancer Center, New York, New York;; 3 Antitumor Assessment Core Facility, Memorial Sloan Kettering Cancer Center, New York, New York;; 4 HHMI, Memorial Sloan Kettering Cancer Center, New York, New York; and; 5 Department of Pharmacology, Weill Cornell Graduate School, New York, New York

**Keywords:** senescence, immuno-PET, pancreatic cancer, aging

## Abstract

Identifying cancer therapy resistance is a key time-saving tool for physicians. Part of chemotherapy resistance includes senescence, a persistent state without cell division or cell death. Chemically inducing senescence with the combination of trametinib and palbociclib (TP) yields several tumorigenic and prometastatic factors in pancreatic cancer models with many potential antibody-based targets. In particular, urokinase plasminogen activator receptor (uPAR) has been shown to be a membrane-bound marker of senescence in addition to an oncology target. **Methods:** Here, 2 antibodies against murine uPAR and human uPAR were developed as immuno-PET agents to noninvasively track uPAR antigen abundance. **Results:** TP treatment increased cell uptake both in murine KPC cells and in human MiaPaCa2 cells. In vivo, subcutaneously implanted murine KPC tumors had high tumor uptake with the antimurine uPAR antibody independently of TP in young mice, yet uPAR uptake was maintained in aged mice on TP. Mice xenografted with human MiaPaCa2 tumors showed a significant increase in tumor uptake on TP therapy when imaged with the antihuman uPAR antibody. Imaging with either uPAR antibody was found to be more tumor-selective than imaging with [^18^F]FDG or [^18^F]F-DPA-714. **Conclusion:** The use of radiolabeled uPAR-targeting antibodies provides a new antibody-based PET imaging candidate for pancreatic cancer imaging as well as chemotherapy-induced senescence.

## Visual Abstract

**Figure:**
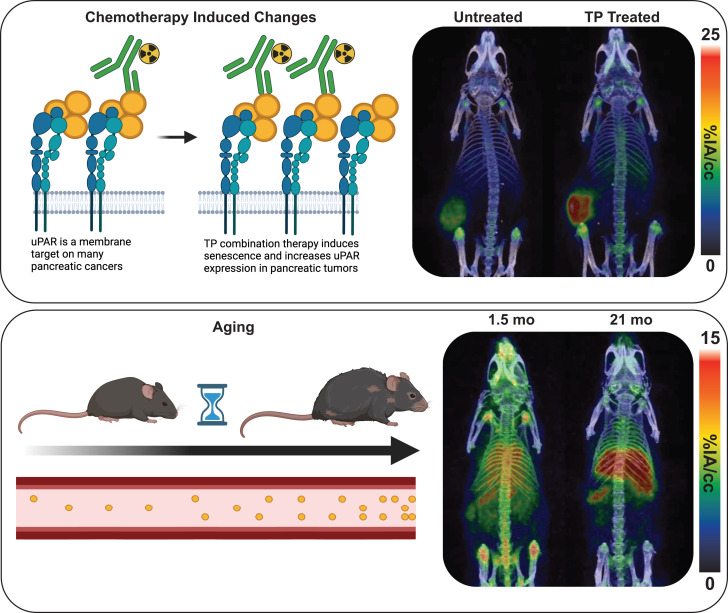

Pancreatic cancer is the fourth leading cause of cancer-related deaths for men and women, with 5-y overall survival rates of around 10% (1). Despite years of cancer research and new immunotherapy solutions, pancreatic cancer is still chemotherapy-focused, starting with gemcitabine in combination with nab-paclitaxel or FOLFIRINOX (folinic acid, 5-fluorouracil, irinotecan, and oxaliplatin) (2). Numerous cytostatic drug combinations induce senescence, a persistent antiapoptotic process characterized primarily by β-galactosidase (β-gal) activity (3,4) with additional markers such as P16^INK4A^ and P21 present (5,6), though not exclusively senescence markers. Furthermore, senescence can be induced through myriad methods such as oncogene activation (7), radiation (8), chemotherapy (9), wound healing (10), or subject age (11,12). Senescence has also been considered a reversible process with cell plasticity (13) and a new hallmark of cancer (14). Senescence molecular imaging has focused on β-gal activity, with a PET version shown in preclinical (15) and recently in clinical trials. Crucial drawbacks to β-gal as a target are the ubiquitous presence of β-gal in lysosomes and pH-dependent activity (16). To improve targeting and image signal-to-noise ratio, membrane-bound antigen–specific PET imaging agents are needed to better quantify senescence in these studies. This work focuses immuno-PET imaging of pancreatic cancer on urokinase plasminogen activator receptor (uPAR) in the presence of the combination of trametinib and palbociclib (TP). TP collectively inhibits mitogen-activated protein kinase kinase and cyclin-dependent kinases 4/6, respectively, in lung (17) and pancreatic (18) cancer models, activating in concert the senescence-associated secretory profile (17–21) for additional shed antigens.

Previously, a chimeric antigen receptor T cell was developed targeting uPAR as a senolytic agent after the induction of senescence by replication or liver damage (22). This work has been advanced recently to include reversal of aging phenotypes as well (23). uPAR is a membrane-bound glycosylphosphatidylinositol-anchored protein that has long been implicated in inflammation and tumor remodeling, with expression of uPAR correlating with poorer outcomes (24). In pancreatic cancer, uPAR is strongly implicated in progression and clinical overall survival (25). Previous immuno-PET (26–29) and AE105 peptide (30)–based PET agents for uPAR have been developed and translated (31,32) to the clinic as imaging agents, but uPAR-targeted imaging has not been used in the context of mouse age or TP-induced senescence.

Here, we characterized 2 immuno-PET antibodies targeting the human uPAR (huPAR) and murine uPAR (muPAR) forms from commercial sources to address preclinical questions in aging, pancreatic cancer, and chemotherapy-induced senescence. In this work, development of an imaging agent for uPAR identifies heterogeneity in pancreatic tumors and cases in which chemically inducing senescence increases uPAR expression. A stronger biologic link to senescence through cell membrane activity is built with specific immuno-PET imaging agents and provides a new senescence-related antigen to tell a more complete story of senescence than before. By establishing these preclinical imaging models, this work will support future antibodies under clinical development and complement additional antigen-based PET agents for other markers of senescence, especially targeting the senescence-associated secretory profile. Ideally, future clinical uPAR imaging agents will provide more specific imaging of pancreatic cancer undergoing therapy and alert clinicians earlier to therapy-refractive disease.

## MATERIALS AND METHODS

Antibodies targeting huPAR (clone 62022, catalog no. MAB807-500) and muPAR (clone 109801, catalog no. MAB531-500) were obtained from R&D Systems with the intention of producing deferoxamine (DFO)–antibody conjugates for radiolabeling with ^89^Zr: [^89^Zr]Zr-DFO-antibodies against huPAR (anti-huPAR) and [^89^Zr]Zr-DFO-antibodies against muPAR (anti-muPAR), respectively. A lysine-reactive *p*-SCN-Bn-DFO (catalog no. B-705) chelator was obtained from Macrocyclics. On the basis of previous methods (33), each antibody was diafiltered with an Amicon Ultra-0.5 30-kD spin filter into Chelex (Bio-Rad)-treated phosphate-buffered saline adjusted to pH 8.7. A 6-molar excess of *p*-SCN-Bn-DFO was added after antibody concentration had been determined by A280 absorbance via nanodrop to be within 2–5 mg/mL, and *p*-SCN-Bn-DFO was added via a dimethylsulfoxide stock solution (1%–2% total dimethylsulfoxide) to the antibody and allowed to mix over 1 h at 37°C on a ThermoMixer (Eppendorf). PD-10 purification was then performed to separate any unreacted chelator, before a final concentration of about 2 mg/mL was achieved via an Amicon spin filter. Apparent specific activities of 462–555 kBq of ^89^Zr per microgram of antibody (10–15 μCi/μg) were observed for in vivo studies with 20 μg injected per mouse, except for the CD1 aging study, which used 10 μg. Cell uptake studies with anti-muPAR and anti-huPAR antibodies were radiolabeled at 370 kBq per microgram of antibody. Radiolabeling was performed via the 4-(2-hydroxyethyl)-1-piperazineethanesulfonic acid neutralization of ^89^Zr-oxalate and subsequent addition of either DFO-anti-uPAR antibody and heating at 37°C for 1 h. Instant thin-layer chromatography analysis was performed in 50 mM ethylenediaminetetraacetic acid, pH 5.5, to determine radiochemical purity, with initial labeling greater than 99% for each experiment (Supplemental Fig. 1A; supplemental materials are available at http://jnm.snmjournals.org). Serum stability was performed once by instant thin-layer chromatography through 144 h with human serum and 3 technical replicates per time point, yielding more than 94% activity bound (Supplemental Fig. 1B). Matrix-assisted laser desorption/ionization time-of-flight mass spectrometry/mass spectrometry was performed (Alberta Proteomics and Mass Spectrometry Facility, University of Alberta) on the immunoconjugates and determined the number of DFO molecules on average per antibody to be 2 and 2.4 chelates for huPAR and muPAR antibodies, respectively. The degree of labeling was determined by the mass difference between conjugated and unconjugated antibodies divided by the molecular weight of DFO. Immunoconjugates were determined by sodium dodecyl sulfate–polyacrylamide gel electrophoresis under nonreducing conditions loading 20 μg of antibody and running for 90 min at 150 V. A Precision Plus dual color ladder was used for size reference (catalog no. 1610374; Bio-Rad). Gel was stained with Imperial stain (catalog no. 24615; Fisher) and imaged on a Licor Odyssey system.

Additional methods can be found in the supplemental materials.

## RESULTS

**FIGURE 1.**
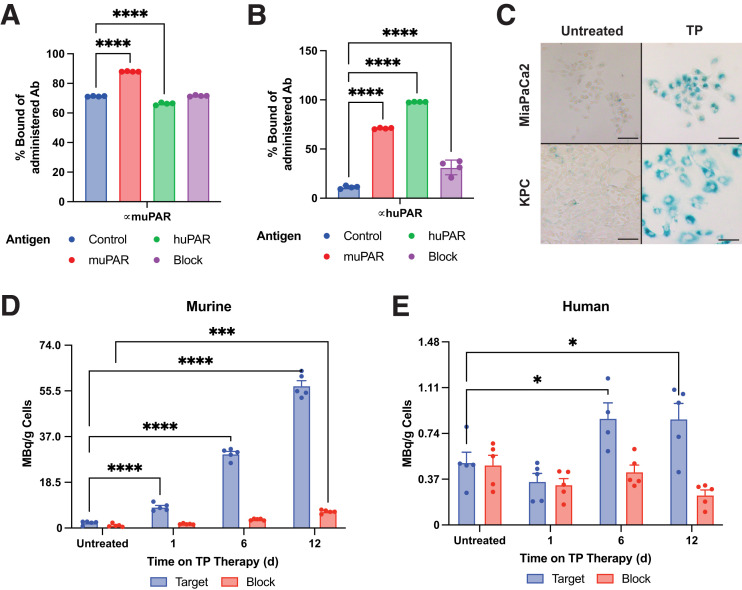
Radiochemistry and target binding of huPAR and muPAR immuno-PET antibodies. (A) Bead binding assay with muPAR- and huPAR-coated magnetic beads via His tag shows high binding of [^89^Zr]Zr-DFO-anti-muPAR to all conditions; still, significant increase in bound activity was found for muPAR antigen beads. Binding was significantly below that of control beads with huPAR antigen, and there was no significant difference from control with blocked beads. (B) Identical bead binding with [^89^Zr]Zr-DFO-anti-huPAR shows better specificity to control beads and significant difference for both muPAR and huPAR antigens, confirming mouse–human cross reactivity of [^89^Zr]Zr-DFO-anti-huPAR antibody. (C) Increased β-gal staining alongside morphologic changes shows that TP induces senescence in KPC and MiaPaCa2 cells. (D) Murine pancreatic cancer line KPC was treated with TP for 1, 6, or 12 d, showing significant increase in [^89^Zr]Zr-DFO-anti-muPAR binding, which can be blocked. (E) Uptake of [^89^Zr]Zr-DFO-anti-huPAR in MiaPaCa2, despite lower activity uptake overall, was significantly increased as well on TP treatment. There were 4 technical replicates in A and B and 5 technical replicates in D and E. Scale bar in C represents 100 μm. *P < 0.05. **P < 0.005. ***P < 0.0005. ****P < 0.0001. Ab = antibody.

Leveraging the muPAR clone used for a chimeric antigen receptor T cell (22,23), we conjugated the muPAR and huPAR antibodies with DFO and subsequently radiolabeled them with ^89^Zr and found them to be suitable for experimentation. In an optimized bead-binding assay (34), [^89^Zr]Zr-DFO-anti-muPAR bound muPAR antigen–functionalized beads the best, though high nonspecific binding was seen with unfunctionalized beads alone (Fig. 1A; Supplemental Fig. 1C). [^89^Zr]Zr-DFO-anti-muPAR binding was also blocked with a 100-fold excess of anti-muPAR antibody, whereas [^89^Zr]Zr-DFO-anti-muPAR binding to huPAR antigen beads was comparable to that to control beads. Antibody preincubation of a 5-fold molar excess of his-tagged muPAR increased bead binding with huPAR domain variants, strongly blocking uptake (Supplemental Fig. 1D). With [^89^Zr]Zr-DFO-anti-huPAR, high uptake in huPAR antigen–coated beads was observed, with significant reductions in control, blocked, and muPAR antigen–containing beads (Fig. 1B). Further examination with huPAR domain fragments identified the best binding with the full 1- to 3-domain sequence, with no binding to huPAR domains 2–3 or 3 alone, suggesting that domain 1 likely contains the epitope for [^89^Zr]Zr-DFO-anti-huPAR and that preincubation studies blocked the huPAR antibody (Supplemental Fig. 1E). Both antibodies exhibited the best binding with the intended antigen species (human–human or mouse–mouse) and could be blocked by an excess of unlabeled antibody; cross reactivity of anti-huPAR and anti-muPAR antibodies was observed as stated by the manufacturer. Both KPC and MiaPaCa2 cells were confirmed to be senescent by β-gal staining over untreated cells after TP treatment for 8 d (Fig. 1C). In a cell-based assay, TP treatment through 12 d significantly increased [^89^Zr]Zr-DFO-anti-muPAR uptake (Fig. 1D), whereas a modest [^89^Zr]Zr-DFO-anti-huPAR increase was observed for the human MiaPaCa2 cell line (Fig. 1E). Altogether, we showed that anti-muPAR and anti-huPAR antibodies can bind their intended target with increased uPAR uptake during TP therapy with cells classically senescent with β-gal staining.

**FIGURE 2.**
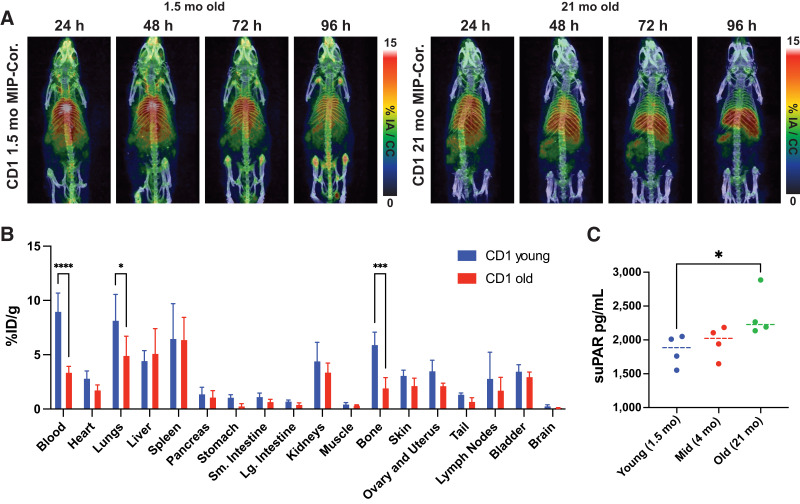
Background imaging of muPAR in healthy CD1 mice shows decrease in antibody distribution and increase in soluble uPAR with age. (A) [^89^Zr]Zr-DFO-anti-muPAR distribution by PET in 1.5-mo-old mice was found to be much more diffuse by 96 h than in geriatric mice at age of 21 mo. (B) Terminal biodistribution confirmed immuno-PET imaging, with significant decrease in radiotracer distribution in geriatric mouse blood, lungs, and bone. (C) Blood from littermates of immuno-PET study at 1.5, 4, and 21 mo of age were assayed by enzyme-linked immunosorbent assay for soluble uPAR, showing significant increase in soluble uPAR between 1.5- and 21-mo-old mice. There were 5 mice per cohort, aged in Memorial Sloan Kettering Cancer Center vivarium, in A and B, and 4 mice per age group with 3 technical replicates per mouse in C. One-way ANOVA statistical analysis was used in C. *P < 0.05. ***P < 0.0005. ****P < 0.0001. %IA = percentage injected activity; %ID = percentage injected dose; bars = median; Cor. = coronal projection; error = SEM; MIP = maximum-intensity projection; suPAR = soluble uPAR.

Previous reports have shown that in addition to membrane-bound uPAR found with age (23), a soluble uPAR component has been shown to increase with age (35) among other factors (36). Older mice showed a decreased distribution of [^89^Zr]Zr-DFO-anti-muPAR, with most clearing to the liver and spleen by PET (Fig. 2A; Supplemental Fig. 2) and terminal biodistribution (Fig. 2B) confirming a significantly lower distribution in blood, lungs, and bone with marrow. In age-matched mice, no significant difference was seen in soluble uPAR levels between the 1.5- and 4-mo-old mice (Fig. 2C) by enzyme-linked immunosorbent assay. In 21-mo-old mice, a significantly higher amount of soluble uPAR was detected in plasma, agreeing with previous reports on patients (36). To minimize differences between groups, mice were age-matched.

As a corollary to uPAR imaging, 1.5- and 21-mo-old mouse cohorts were imaged beforehand with the macrophage translocator protein translator protein tracer *N,N*-diethyl-2-[4-(2-fluoroethoxy)phenyl]-5,7-dimethylpyrazolo[1,5-a]pyrimidine-3-acetamide ([^18^F]F-DPA-714) for inflammation (37,38), for which translocator protein expression is increased on inflammation from a variety of sources. We hypothesized that [^18^F]F-DPA-714 might quantify changes in inflammation in age or after chemotherapy-induced senescence. However, no tumor uptake difference was seen by age (Supplemental Fig. 3), as the MiaPaCa2 tumor–bearing model was exclusive of radiotracer uptake (Supplemental Fig. 4) in agreement with previous tumor models (39).

**FIGURE 3.**
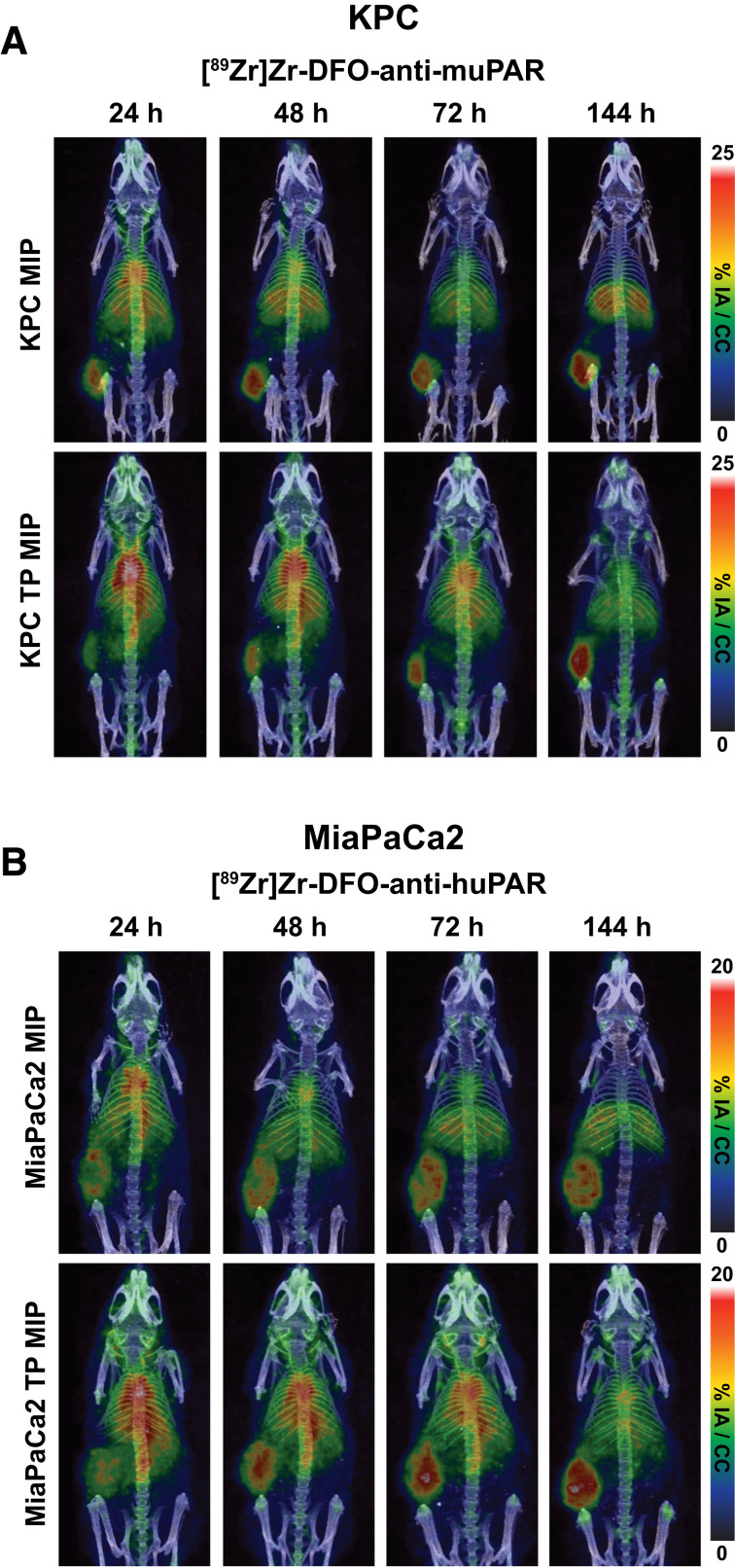
With greater specificity than [^18^F]FDG, immuno-PET of muPAR and huPAR showed that human and murine pancreatic cancer increased with senescence-inducing therapy. (A) [^89^Zr]Zr-DFO-anti-muPAR PET imaging at 24–144 h in KPC-bearing mice showed clear uptake in tumor with and without TP treatment, though with reduced distribution in liver and increased uptake in blood after TP treatment. Terminal biodistribution can be found in Supplemental Figure 5A. (B) [^89^Zr]Zr-DFO-anti-huPAR PET imaging at 24–144 h in MiaPaCa2 showed lower tumor targeting in untreated mice than did [^89^Zr]Zr-DFO-anti-muPAR imaging. On TP treatment, tumor uptake increased significantly with TP, as seen in terminal biodistribution (Supplemental Fig. 5B), and was more specific to tumor than with [^18^F]FDG imaging (Supplemental Fig. 5D). There were 5 mice per group. Region-of-interest analysis of heart, liver, and tumor can be found for KPC and MiaPaCa2 models in Supplemental Figure 6. %IA = percentage injected activity.

**FIGURE 4.**
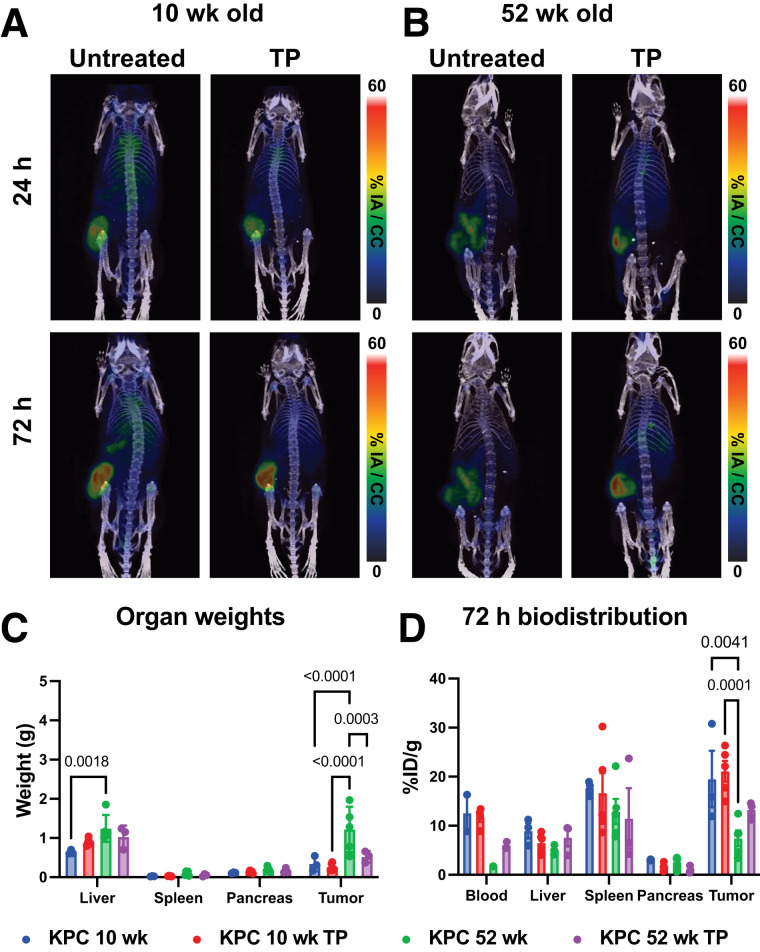
[^89^Zr]Zr-DFO-anti-muPAR targeting of 10- and 52-wk-old mice bearing KPC flank tumors on or off TP therapy. (A) Ten-week-old mice bearing flank KPC tumors showed rapid accumulation of [^89^Zr]Zr-DFO-anti-muPAR in tumor after 24 h and through 72 h, with no discernable difference between untreated and TP-treated tumors. (B) Mice aged 52 wk that were implanted and imaged at same time as mice in A showed less uptake of [^89^Zr]Zr-DFO-anti-muPAR in untreated group with increase in TP treatment. (C) In 52-wk-old untreated mice, larger tumors were seen than in 10-wk-old mice, as expected for mice with advanced age and diminished immune capacity. No other differences in gross organ findings between 10 and 52 wk old were observed other than enlargement of liver in aged mice. (D) Terminal biodistribution at 72 h shows decrease in [^89^Zr]Zr-DFO-anti-muPAR in blood as seen in Figure 2C for aged mice, and significant decrease in tumor targeting was also seen for untreated mice. TP-treated mice had slightly higher average uptake values, but differences were not statistically significant. Although [^89^Zr]Zr-DFO-anti-muPAR rapidly identified KPC flank tumors, TP treatment did not yield significant increase in tumor uptake in either TP group; tumor size was same as that of mice 10 wk old. %IA = percentage injected activity; %ID = percentage injected dose.

To identify uPAR in vivo alongside TP treatment, 5-mo-old c57bl6j mice bearing KPC flank tumors were injected with 40 μg of [^89^Zr]Zr-DFO-anti-muPAR and imaged at 24, 48, 72, and 144 h after intravenous injection. KPC tumors were uPAR-avid, with no significant increase in uptake while on TP treatment by PET (Fig. 3A) and confirmed by terminal biodistribution (Supplemental Fig. 5A). Region-of-interest analysis of heart, liver, and tumor for KPC mice identified similar uptake kinetics, though with higher blood values for TP-treated mice (Supplemental Fig. 6) and tumor uptake for TP-treated mice (9.8 vs. 4.7 μg of antibody/g of tumor). For comparison, imaging of the same mice 2 wk earlier with [^18^F]FDG showed less specific uptake by the tumor (Supplemental Fig. 5C), and nude mice implanted with KPC tumors recapitulated the in vitro results, with significantly higher tumor uptake of [^89^Zr]Zr-DFO-anti-muPAR (Supplemental Fig. 7B). To take age into consideration, muPAR PET imaging was repeated with 10- and 52-wk-old c57bl6j mice bearing KPC tumors implanted at the same time (Figs. 4A and 4B; all mice, Supplemental Fig. 8). Ten-week-old mice had no discernable difference in [^89^Zr]Zr-DFO-anti-muPAR uptake (Fig. 4A), whereas uptake was significantly reduced in 52-wk-old non–TP-treated mice (Fig. 4B). Although aged and untreated mice had larger tumors, possibly from a weakened immune system (40), 52-wk-old TP-treated mice had tumor sizes indistinguishable from those of 10-wk-old untreated mice (Fig. 4C). In all age cohorts for KPC-bearing c57bl6j mice, [^89^Zr]Zr-DFO-anti-muPAR at all ages rapidly identified tumors, yet there was no significant difference in uPAR antibody uptake with TP, even though the average was higher (Fig. 4D).

Switching to the anti-huPAR antibody, PET imaging showed a greater distribution into the liver and spleen as predicted for increased immunocompromised animal models (41); however, a clearer difference between untreated and TP-treated tumors by 144 h after injection was observed (Fig. 3B), with different uptake kinetics for the TP versus untreated tumor uptake, with again more radiotracer in the blood pool for TP-treated mice (Supplemental Fig. 6) and a higher receptor-binding capacity with TP treatment (4.0 vs. 2.4 μg of antibody/g of tumor). Terminal biodistribution confirmed a significant difference in tumor uptake between untreated and TP-treated mice (Supplemental Fig. 5B), showing that uPAR changes can be visualized with TP treatment over already-present uPAR expression. Most important, prior [^18^F]FDG imaging did not identify the tumor (Supplemental Fig. 5D). Immunohistochemistry of untreated and TP-treated tumors showed Ki-67, P21, P16, uPAR, and β-gal staining in sequential slides (Supplemental Fig. 9), revealing heterogeneity in each tumor—particularly uPAR and β-gal staining as adjacent stains—and uPAR staining closely matching Ki-67 staining patterns. Of note, P21 and P16, which are classic markers of senescence, were absent from MiaPaCa2 tissue because of the homozygous deletion of CDKN2A in the MiaPaCa2 line (42), and the lack of P21 staining was likely due to intermittent instead of constant TP dosing (43).

We next looked to optimize tumor delivery. Anti-huPAR antibody doses in the range of 10–20 μg yielded the highest tumor uptake, whereas preloading in addition to 40-μg doses yielded lower tumor uptake (Supplemental Figs. 10A and 10B). Using 20 μg of [^89^Zr]Zr-DFO-anti-huPAR plus a combination of nonspecific IgG_1_ and huPAR-specific block, only the huPAR antibody blocked tumor uptake (Supplemental Fig. 11A), whereas IgG_1_ blocking lowered off-target organ uptake as confirmed by terminal biodistribution (Supplemental Fig. 11B) alongside PET region-of-interest analysis (Supplemental Fig. 6). In addition, the timing of TP administration (day 0, −1, or −2) and duration (1, 2, or 4 doses) revealed (Supplemental Fig. 12A) that 1 dose the same day as the radiotracer was significantly higher for tumor uptake while lower for liver accumulation (Supplemental Fig. 12B). Thus, future therapy studies on young mice could implement TP at the time of imaging instead of a week before, though for aged mice TP treatment kept tumor sizes dramatically lower.

## DISCUSSION

Overall, we have characterized and developed 2 uPAR immuno-PET molecular imaging agents for preclinical studies, showing selectivity, cross reactivity, and binding regions of both antibodies tested as oncology and senescence imaging agents. Distinction will require additional markers, potentially with multiplexed PET (44) to improve quantification of senescence. This work also relied on commercially sourced antibodies for development with the possibility for batch-to-batch variation beyond antibody concentration. More human-specific uPAR antibodies focused on nonshed epitopes will be important for clinical accuracy. In addition, we have confirmed in mice the increase in soluble uPAR with age, with clear differences in biodistribution between young and elderly mice. However, in many aging studies, conditions such as housing and age-related diseases need to be further considered in any interpretation (45–47). In young, age-matched mice, immuno-PET with [^89^Zr]Zr-DFO-anti-muPAR readily identified the murine pancreatic flank KPC tumor. Furthermore, [^89^Zr]Zr-DFO-anti-huPAR uptake was significant between TP-treated and untreated groups only at the lowest, 10-μg, dose, with minor improvement using an IgG_1_ block to off-target organs. Lastly, in KPC-bearing mice a single dose of TP was as effective as 4 prior doses. By building on known senescence models using KPC and MiaPaCa2 cell lines undergoing TP treatment (17,18), uPAR immuno-PET can be used as new cancer or senescence tools.

## CONCLUSION

Although senescence is multifaceted, with genetic, chemotherapeutic, and replicative origins, imaging of senescence has relied solely on β-gal activity to confirm presence. Building molecular imaging agents that target discrete antigens will improve understanding of the local and global effects of senescent tissue, enabling physicians and researchers to see therapy resistance. In this work, the senescence-inducing TP significantly increased uPAR antibody uptake by PET in vitro as well as in a few in vivo models above that of basal uPAR expression found in pancreatic cancer tissue. uPAR as a membrane-bound antigen also was confirmed to increasingly be shed with age, and a nonspecific blocking strategy improved tumor targeting overall. Alone, uPAR is a promising theranostic target for pancreatic cancer, and this work with quantifiable and commercially available immuno-PET agents adds applications in senescence and age.

## DISCLOSURE

This work was supported by the following grants: National Institutes of Health R35 CA232130–04 (to Jason Lewis), S10 OD016207-01 (to Pat Zanzonico, MSKCC), and P30 CA08748 (to Selwyn Vickers, MSKCC). Edwin Pratt was supported by National Institutes of Health grant F32 CA268912-01 and is currently supported by K99 CA276804. Scott Lowe has equity in a joint venture developed by MSKCC and a cell therapy company to develop senolytic cell-based therapies for noncancer indications. The company has also licensed MSKCC IP, including some huPAR binders. The Mark Foundation provided the Endeavor Award to Scott Lowe for “Harnessing Senescence Biology for Immune Oncology.” No other potential conflict of interest relevant to this article was reported.

## Notes

KEY POINTS **QUESTION:** Can immuno-PET tracers be used to quantify chemotherapy-induced senescence and reveal new radiopharmaceutical therapeutic targets? **PERTINENT FINDINGS:** uPAR antibody uptake is increased when the pancreatic cancers tested are administered a senescence-inducing TP signifying uPAR as both an oncology target and a senescence target. Mice with advanced age had higher soluble uPAR values than—and a significantly different biodistribution from—young mice with and without pancreatic tumors. **IMPLICATIONS FOR PATIENT CARE:** Chemotherapy resistance in pancreatic cancer can include senescent cell populations. By noninvasively identifying these populations by PET, physicians can glean therapy resistance earlier by PET avidity and may, in the future, provide uPAR-targeted therapy to remove resistant populations.
